# Bidirectional treatment of peritoneal metastasis with Pressurized IntraPeritoneal Aerosol Chemotherapy (PIPAC) and systemic chemotherapy: a systematic review

**DOI:** 10.1186/s12885-020-6572-6

**Published:** 2020-02-10

**Authors:** Magnus Ploug, Martin Graversen, Per Pfeiffer, Michael Bau Mortensen

**Affiliations:** Odense PIPAC Center (OPC) and Odense Pancreas Center (OPAC), Upper GI and HPB Section, Department of Surgery, Odense University Hospital, J.B.Winsløvs Vej 4, 5000 Odense C, Denmark

**Keywords:** PIPAC, Bidirectional, Peritoneal metastasis, Intraperitoneal chemotherapy, Carcinomatosis

## Abstract

**Background:**

Pressurized intraperitoneal aerosol chemotherapy (PIPAC) is used in the palliative treatment of peritoneal metastasis. The combination of intraperitoneal and systemic chemotherapy seems rational, and the aim of this systematic review was to compare PIPAC directed monotherapy with a bidirectional treatment approach (PIPAC in combination with systemic chemotherapy). Main outcomes were survival and quality of life.

**Methods:**

A systematic literature search in Medline, Embase, Cochrane and the “Pleura and Peritoneum” was conducted and analyzed according to PRISMA guidelines. Studies in English reporting on bidirectional treatment with PIPAC and systemic chemotherapy and published before April 2019 were included.

**Results:**

Twelve studies with a total of 386 patients were included. None were specifically designed to compare mono- versus bidirectional treatment, but 44% of the patients received bidirectional treatment. This was more frequent in women (non-gynecological cancers) and one-third of the bidirectional treated patients had received no prior chemotherapy. Data from the included studies provided no conclusions regarding survival or quality of life.

**Conclusion:**

Bidirectional treatment with PIPAC and systemic chemotherapy is practised and feasible, and some patients are enrolled having received no prior systemic chemotherapy for their PM.

The difficulty in drawing any conclusions based on this systematic review has highlighted the urgent need to improve and standardize reports on PIPAC directed therapy. We have, therefore, constructed a list of items to be considered when reporting on clinical PIPAC research.

**Trial registration:**

International Prospective Register of Systematic Reviews, PROSPERO. Registration number: 90352, March 5, 2018.

## Background

Pressurized intraperitoneal aerosol chemotherapy (PIPAC) is a novel approach in the palliative treatment of non-resectable peritoneal metastasis (PM) based on laparoscopically administered, aerosolized chemotherapy into the hyperbaric capnoperitoneum [1, 2]. Compared to systemic chemotherapy, PIPAC provides significantly higher concentrations of chemotherapeutics in the peritoneum, but a low concentration in the systemic circulation [3, 4] thereby, avoiding adverse effects from systemic administration. Since intravenous chemotherapy may enhance drug accumulation in the subperitoneal space and simultaneously have a direct effect on systemic micro-metastasis, the combination of intraperitoneal and systemic chemotherapy (i.e. bidirectional treatment) is a rational approach to treatment in patients with PM [4]. The bidirectional treatment approach might lead to a new mainstay in palliative cancer treatment, where the disseminated malignancy is targeted from two directions instead of one.

The idea of combining intraperitoneal and systemic chemotherapy is not new, and both treatments may be delivered in several different ways. Hyperthermic intraperitoneal chemotherapy (HIPEC) - introduced roughly 30 years before the first clinical PIPAC data [3, 5], is one method of delivering intraperitoneal chemotherapy (IP). Combined with cytoreductive surgery, HIPEC provides a potentially curative strategy in selected patients with PM [6, 7]. Although trials on the use of PIPAC directed treatment as prophylaxis for intraperitoneal metastases are currently in progress, PIPAC is, to date, considered a palliative treatment. Through PIPAC, intraperitoneal chemotherapy can be delivered in a safe and repeatable way without systemic adverse effects. Objective local tumor response rates to PIPAC above 50% have been reported in patients with PM from a wide variety of primary cancers [8], without compromising the patient’s quality of life (QoL) [8]. Bidirectional treatment strategies, not involving PIPAC, have been used and studied extensively and have shown improvement in overall and progression free survival compared to intraperitoneal treatment alone in patients with advanced ovarian or gastric cancers [9–12].

Based on these observations, a bidirectional approach combining PIPAC and systemic chemotherapy seems logical. Systemic chemotherapy, however, is associated with significant toxicity [13] and, a recent prospective multicenter study on patients with end-stage cancer, found that it did not improve QoL for patients with moderate and poor performance status. Furthermore, systemic chemotherapy had a significant deteriorating effect on QoL for patients with a good performance status [14]. In 2012, the American Society of Clinical Oncology advised against palliative chemotherapy for solid tumor malignancies in patients with poor performance status and no prior benefit from systemic treatment [15]. Hence, if systemic chemotherapy is introduced between PIPAC procedures, it is of great importance to monitor not only performance status and survival, but also QoL, complications and adverse events.

The primary aim of this systematic review was to investigate if bidirectional treatment with PIPAC plus systemic chemotherapy led to higher QoL scores and better survival than PIPAC alone in patients with PM of any origin. The secondary aim was to evaluate additional outcome variables in a bidirectional treatment setting including potential complications/adverse events and objective tumor response.

### Definition

The term “bidirectional” in the delivery of chemotherapy is widely used but no clear or consensus definition exists [16–20]. Put simply, “bidirectional” means “operating or functioning in two (usually opposite) directions”. With respect to chemotherapy, we define “bidirectional” as when two different administration routes are used in combination. Thus, in this review, bidirectional treatment describes when PIPAC is used in combination with systemic chemotherapy as opposed to monodirectional treatment where PIPAC is the only route of administration. In this definition, we included regimens where systemic chemotherapy was delivered either on the same day as PIPAC, between PIPAC procedures or in a continuous fashion during the PIPAC course (e.g. oral chemotherapy). We excluded treatment regimens where systemic chemotherapy was only given prior to the first PIPAC procedure, after the last PIPAC procedure or as a combination of the two strategies. In general, the term “bidirectional” should not be limited to a specific combination of administration routes but should rather be used to state that more than one route of administration is used in targeting the malignancy. This could be intravenous (IV) chemotherapy combined with IP (whether it is delivered as PIPAC, HIPEC, catheter-based or others) but also describes combinations including intraluminal chemotherapy or heretofore undiscovered ways of administering chemotherapy. Bidirectional chemotherapy, therefore, must always be accompanied by details on the routes of administration.

## Methods

This review was performed according to the Preferred Reporting Items for Systematic Reviews and Meta-Analyses (PRISMA) guidelines [21]. A review protocol following the PRISMA-P guidelines [22] was published at the International Prospective Register of Systematic Reviews (PROSPERO - registration number: 90352) on March 5, 2018. Studies on adult patients with PM, written in English, and with reports of a bidirectional treatment regimen with PIPAC and systemic chemotherapy were considered for inclusion. No publication status restrictions were imposed, and only review articles and books/book chapters were excluded.

Studies were identified through a literature search in Medline (OVID interface, 1946-present), Embase (OVID interface, 1979-present) and in the Cochrane Central Register of Controlled Trials (CENTRAL). In addition, we manually searched the journal “Pleura and Peritoneum – De Gruyter” (all issues to date) and scanned the reference lists of included studies or relevant systematic reviews.

To ensure literature saturation, an additional search was performed immediately prior to the submission of this review using the same search string but limited to prospective trials with the primary objective of comparing bidirectional to monodirectional PIPAC. To our knowledge, the first in-human application of intraperitoneal pressurized chemotherapy was performed in 2011 [3], and, therefore, our search was limited to the time period of 2011 to present. The specific search strategies were created in collaboration with a Health Sciences Librarian who has expertise in systematic review searching. We searched “Title and Abstracts” based on the following strategy (Medline search - Ovid interface)

1. PIPAC.ti,ab.
2. exp. Antineoplastic Agents/
3. chemotherap*.ti,ab.
4. 2 or 3
5. (capnoperito* or pneumoperito* or pressur*).ti,ab.
6. (abdom* or intra-abdom* or intraabdom* or perito* or intraperito* or intra-perito*).ti,ab.
7. 4 and 5 and 6
8. 1 or 7
9. Limit 8 to (english language and humans and yr = “2011 -current”)

The citations were uploaded to the COVIDENCE Software, (www.covidence.org), facilitating the selection process which was performed independently by two reviewers (MP and MG). In phase one of the selection process, titles and abstracts were screened and those that met the eligibility criteria (or where it was thought possible that a full text examination would find it to do so) were included. Subsequently, full text articles from all eligible works were examined, in detail. Disagreements between reviewers were resolved by consensus or, if consensus could not be achieved, a pre-designated third reviewer (MBM) made the final decision on the inclusion of the article.

In order to evaluate the results after bidirectional therapy, overall survival (OS), progression free survival (PFS) and QoL were noted as main outcome variables. In addition, the performance status (PS) during inclusion, number of PIPAC procedures per patient, interval between PIPAC procedures, surgery related complications, adverse events and objective tumor response (OTR) were registered. Additionally, study details (author, title, publication year, study type), demographic information (age, gender, type of malignancy, time from diagnosis of PM to first PIPAC, previous treatment), intervention details (type and dosage of chemotherapy, ratio between mono- and bidirectional treated patients, frequency and duration of treatment) and finally any descriptive data with reasoning or comments on the bidirectional approach were included.

Data extraction was done independently by two reviewers (MP and MG) with disagreements resolved in the same manner as in the selection process.

If one of the reviewers suspected that data might be in duplicate, overlapping or reported in companion studies, this was evaluated by the group of authors. Publications from the same geographic location or where two or more authors were identical were examined for possible duplication. The risk of bias in individual studies and the risk of meta-bias(es) were also assessed.

## Results

**Fig. 1:**
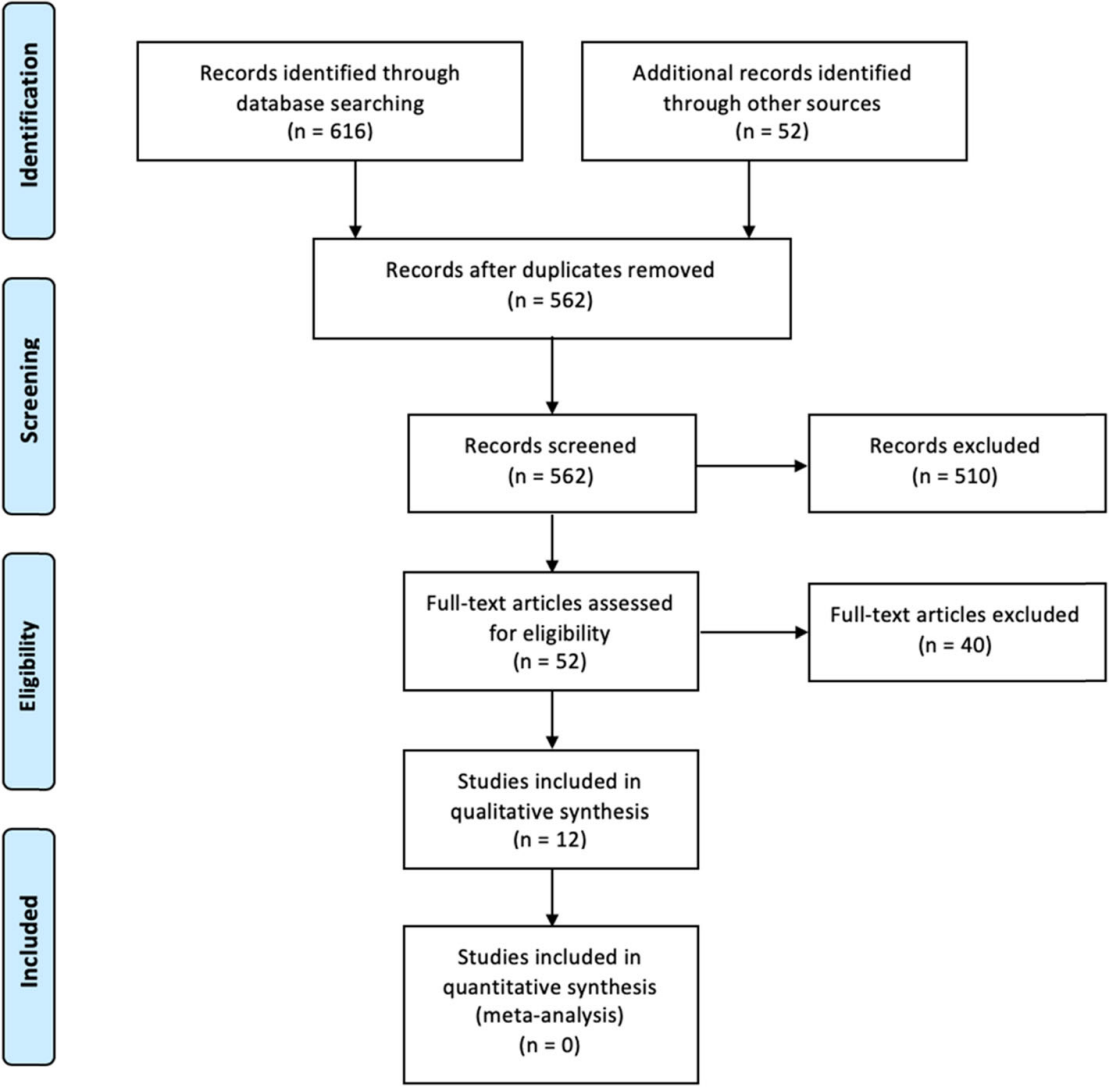
Prisma flow chart [21]

The primary literature search was conducted on March 6, 2018 (Fig. 1). The search of Medline, Embase, and The Cochrane Central Register of Controlled Trials yielded 616 citations while 52 citations were found in the journal “Pleura and Peritoneum”. After adjusting for duplicates, 562 remained. 510 studies were excluded after reviewing the abstracts. The full text of the remaining 52 citations was examined and a further 38 citations were excluded, leaving 14 publications relevant for inclusion. No additional studies were found through the reference lists of included studies or from relevant systematic reviews. Upon careful examination of the 14 publications, two conference abstracts were found to possibly represent the same patients as reported in included articles. The abstract by Khomyakov et al. [23] was removed after contacting the author to confirm it represented the same patients as in their article [24]. The conference abstract by Robella et al. [25] was excluded due to convincing overlap in timeframe, author group and location with their included article [26], and due to the lack of any numerical data on bidirectional treatment. Thus, a total of 12 publications were included in the final analysis. The literature search was repeated on April 7th, 2019, but no additional studies were identified.

All publications were non-randomized and descriptive, having no control group and none were designed to compare bidirectional to monodirectional treatment.

There were 386 patients, in total, who were entered into a PIPAC program. Information on the distribution between mono- and bidirectionally treated patients was available in 326 patients, with 44% receiving bidirectional treatment (*n* = 145) (Table 1). Data on the type of malignancy in combination with the type of treatment (mono- vs. bidirectional) was reported in 211 patients and 38% (*n* = 80) of these received bidirectional treatment with the following malignancy specific variations: gynecological 10% (9/87), pancreatic 27% (7/26), biliary tract 29% (4/14), mesothelioma 50% (1/2), colorectal 68% (13/19), gastric 74% (45/61), pseudomyxoma 100% (1/1) and cancer of unknown primary (CUP) 0% (0/1) (Table 1).

**Table 1:** Study characteristics and demographics

Author and publication year	Design	Malignancies	Number of patients	Number of patients with Bidirectional treatment		Gender (male/female)	Age, years (range or SD)	Previous tumor related surgery	Previous systemic chemotherapy
Alyami et al. 2017 [27]	Retrospective	Total	73	64	Total:	31/42	57.1 (32.3-77.9)^d^	NR	64
		- Gastric	26	NR	Bidirectional:	NR	NR	NR	NR
		- Colon	20	NR					
		- Ovarian	13	NR					
		- Mesothelioma	8	NR					
		- Pseudomyxoma	1	NR					
		- Others	5	NR					
Demtröder et al. 2015 [28]	Retrospective	Colorectal	17	11	Total:	10/7	59 (+/-12)^e^	17	16
					Bidirectional:	NR	NR	11	10
Falkenstein et al. 2018 [29]	Retrospective	Biliary tract	13	3	Total:	8/5	58 (37-75)^f^	9	7
					Bidirectional:	NR	NR	NR	NR
Graversen et al. 2017 [30]	Prospective	Pancreatic	5	1	Total:	3/2	62 (49-69)^g^	2	5
					Bidirectional:	0/1	62	0	1
Hilal et al. 2017 [31]	Retrospective	Total (all gynecological)	84	6	Total:	0/84	60.4 (+/-12.2)^e^	84^i^	84
		- Ovarian	77	NR	Bidirectional:	0/6	NR	6^i^	6
		- Fallopian tube	2	NR					
		- Peritoneal	5	NR					
Khomyakov et al. 2016 [24]	Prospective	Gastric	31	31	Total:	9/22	52 (25-70)^f^	NR	7
					Bidirectional:	9/22	52 (25-70)^f^	NR	7
Khosrawipour et al. 2017 [32]	Retrospective	Pancreatic	20	6	Total:	15/5	64.9 (45-87)^g^	8	20
					Bidirectional:	5/1	67.5 (52-75)^g^	3	6
Nadiradze et al. 2015 [33]	Retrospective	Gastric	24	8	Total:	12/12	56 (+/-13)^e^	14	19
					Bidirectional:	NR	NR	NR	NR
Reymond et al. 2016 [34]^a^	Retrospective	Total	3	1^b^	Total:	1/2	68 (59-72)^g^	2	3
		- CUP	1	0	Bidirectional:	0/1	59	1	1
		- Pancreatic	1	0					
		- Gallbladder	1	1					
Robella et al. 2016 [26]	Retrospective	Total	14	13	Total:	NR^c^	52.5 (39-78)^g^	9	14
		- Mesothelioma	2	1	Bidirectional:	NR^c^	52 (39-78)^g^	8	13
		- Ovarian	3	3					
		- Colorectal	2	2					
		- Pseudomyxoma	1	1					
		- Gastric	6	6					
Farinha et al. 2017 [35]	Retrospective	Total	42	1	Total:	8/34	66 (59-73)^h^	NR	NR
		- Gynecological	21	NR	Bidirectional:	NR	NR	NR	NR
		- CRC	14	NR					
		- Gastric	3	NR					
		- Small bowel	1	NR					
		- Appendix	1	NR					
		- Pseudomyxoma	1	NR					
		- Mesothelioma	1	NR					
Hübner et al. 2017 [36]	Retrospective	Total	60	NR	Total:	NR	NR	NR	NR
		- Gynecological	NR	NR	Bidirectional:	NR	NR	NR	NR
		- Digestive	NR	NR					

*CUP* cancer of unknown primary, *NR* not reported

^a^Including conventional PIPAC and Electrostatic Precipitation PIPAC (ePIPAC)

^b^Authors stated that all patients received concomitant systemic chemotherapy. Following our definition two patients did not receive bidirectional therapy since systemic chemotherapy was terminated before the first PIPAC procedure

^c^Including three gynecological cancers – all receiving bidirectional treatment

^d^Median with range

^e^Mean with Standard Deviation

^f^Mean with range

^g^Calculated median with range

^h^Median with Inter-Quartile Range

^i^This study concerned relapse of cancer and patients were interpreted as having had primary surgery

From the available data on non-gynecological cancers we found that 50% of the men (*n* = 14/28) and 81% of the women (*n* = 25/31) received bidirectional treatment (Table 1).

Mean and median age ranged from 52 to 68 years. From four studies [26, 30, 32, 34] reporting raw data, the calculated median age was 62 years (*n* = 21, inter-quartile range (IQR) 61–69) in the monodirectional group and 59 years (n = 21, IQR 51–68) in the bidirectional group (Table 1).

Performance status (PS), − either Eastern Cooperative Oncology Group (ECOG) or Karnofsky Index (KI), was reported as a criteria for entering the PIPAC program in six studies, and patients were excluded if PS(ECOG) was > 1 [27], > 2 [24, 26, 30], > 3 [29], or if PS(KI) was < 50% [29, 32]. Two studies stated that poor PS was not an exclusion criterion [28, 33]. The actual PS was reported in five studies as a mean or median KI between 70 and 85% [28, 29, 31–33] with no separation between mono- and bidirectionally treated patients.

Previous tumor related surgery with primary tumor resection was performed in 145 out of 180 patients where data were available. Based on additional information in 143 of these patients 89% (*n* = 93/105) belonged to the monodirectional and 76% (*n* = 29/38) to the bidirectional treatment group (Table 1).

Information on prior systemic chemotherapy was available in 10 publications, and 239 out of 284 patients (84%) had received prior systemic chemotherapy when entering the PIPAC program. Not all publications specified this into mono- or bidirectional treated patients, but when this was specified, all monodirectional treated PIPAC patients (*n* = 105), but only two thirds of the patients receiving bidirectional therapy (44/69), had been pretreated with systemic chemotherapy (Table 1). The duration from diagnosis of PM to the first PIPAC treatment was noted in two trials, and reported as six and 2 months, respectively [29, 34].

The type of chemotherapy used for the PIPAC procedure was either Cisplatin/Doxorubicin or Oxaliplatin with one exception [27] where Mitomycin-c was used in six PIPAC procedures. Numerous different drugs were used for the systemic part of the bidirectional therapy, but with no clear description of the rationale behind their use [24, 26, 30, 32, 34]. Only two papers reported on the number of systemic chemotherapy cycles [30, 32]. The interval between PIPAC procedures was noted in 11 studies and reported to be either 6 weeks [24, 26, 28, 29, 32–36] or four to six weeks [30, 31] (Table 2).

**Table 2:** Intervention details

Author	Malignancies	PIPAC Chemo	Systemic Chemo	Interval between PIPAC
Alyami et al. [27]	Gastric, Colon, Ovarian, Mesothelioma, Pseudomyxoma and others	Oxa, C/D or mito-c	NR	NR
Demtröder et al. [28]	Colorectal	Oxa	NR	6 weeks^a^
Falkenstein et al. [29]	Biliary tract	C/D	NR	6 weeks^a^
Graversen et al. [30]	Pancreatic	C/D	Gem + S-1	4–6 weeks^a^
Hilal et al. [31]	Gynecological	C/D	NR	4–6 weeks^a^
Khomyakov et al. [24]	Gastric	C/D	XELOX	6 weeks^a^
Khosrawipour et al. [32]	Pancreatic	C/D	Gem+nab-Pax Folfirinox Gem	6 weeks^a^
Nadiradze et al. [33]	Gastric	C/D	NR	6 weeks^a^
Reymond et al. [34]^a^	CUP, Pancreatic and Gallbladder	C/D	Cis + Gem	6 weeks^a^
Robella et al. [26]	Mesothelioma, Ovarian, Colorectal, Pseudomyxoma and Gastric	C/D or Oxa	Topotecan Folfox+cetuximab Folfoxiri Paclitaxel Folfiri Paclitaxel+Ramcirumab Xelox Paclitaxel Pemetrexed	6 weeks^a^
Farinha et al. [35]	Gynecological, Colorectal, Gastric, Small bowel, Appendix, Pseudomyxoma and Mesothelioma	NR	NR	6 weeks^a^
Hübner et al. [36]	Gynecological and Digestive	C/D or OXA	NR	6 weeks^a^

*C/D* Cisplatin/Doxorubicin, *CUP* cancer of unknown primary, *gem* Gemcitabine, *mito-c* Mitomycin c, *nab-pax* nab-Paclitaxel, *NR* not reported, *Oxa* Oxaliplatin,

^a^ Pursued rather than actual interval

Overall survival, with a known starting point and with separate data on the mono- and bidirectional group, was only reported in eight patients (two treated bidirectionally) (Table 3).

**Table 3:** Main outcome variables

Author	Reported overall survival (yes/no)	Separately reported overall survival on mono- and bidirectional treated patients yes/no (number of patients)	PFS reported (yes/no)	QoL reported (yes/no)
Alyami et al. [27]	No	No	No	No
Demtröder et al. [28]	Yes (from 1st PIPAC)	No	No	No
Falkenstein et al. [29]	Yes (from 1st PIPAC)	No	No	No
Graversen et al. [30]	Yes (from 1st PIPAC)^a^	Yes (n = 5)	No	No
Hilal et al. [31]	No	No	No	No
Khomyakov et al. [24]	Yes (not clear from when)	Yes (*n* = 31)^c^	No	No
Khosrawipour et al. [32]	Yes (from 1st PIPAC)	No	No	No
Nadiradze et al. [33]	Yes (from 1st PIPAC)	No	No	No
Reymond et al. [34]^a^	Yes (from 1st PIPAC)^b^	Yes (n = 3)	No	No
Robella et al. [26]	No	No	No	Yes
Farinha et al. [35]	No	No	No	Yes
Hübner et al. [36]	No	No	No	No

*PFS* progression free survival, *QoL* Quality of life

^a^Survival also reported from the time of primary tumor resection, and from the time of the PM diagnosis

^b^Survival also reported from the time of diagnosis (of either the primary cancer or of PM)

^c^All patients treated bidirectionally

QoL was reported in two studies (Table 3). One study [26] did not mention it in the methods section and wrote in the results section “QoL was recorded routinely in all patients before the enrolment and after each PIPAC-procedure through two questionnaires: SF-36 and EORTC QLQ-30. No further deterioration of physical, emotional and cognitive scores during therapy were recorded”, but no data was presented. In the study by Farinha et al. [35], the main outcome was QoL assessed with the QLQ-C30 questionnaire but no comparison between mono- and bidirectional treated patients was made.

The median number of PIPAC procedures was 2 (IQR 1–3, *n* = 200), calculated on raw data reported in nine studies (Table 4). The median number of PIPAC procedures in the bidirectional group was 2 (IQR 1–3, *n* = 52). Removing these 52 patients from the combined group the median number of PIPAC treatments remained 2 (IQR 1–3, *n* = 148) (Table 4).

**Table 4:** Additional outcome variables

Author	Median number of PIPAC procedures (number of patients)	Median number of PIPAC procedures in bidirectional treated patients	CTCAE Reporting	Total	Mono	Bidirectional	Objective tumor response reported	Scale used to report objective tumor response
Alyami et al. [27]	2 (*n* = 73)^a^	NR	CTCAE reported	Yes^c^	No	No	Yes	PCI
			Time frame (days)	30				
			Grade 1-2	NR				
			Grade 3-4	16				
			Grade 5	5				
Demtröder et al. [28]	3 (n = 17)^a^	NR	CTCAE reported	Yes^d^	No	No	Yes	TRG
			Time frame (days)	NR				
			Grade 1-2	12				
			Grade 3-4	4				
			Grade 5	0				
Falkenstein et al. [29]	1 (*n* = 13)^a,b^	NR	CTCAE reported	Yes^d^	No	No	Yes	TRG, PCI
			Time frame (days)	NR				
			Grade 1-2	9				
			Grade 3-4	0				
			Grade 5	0				
Graversen et al. [30]	3 (*n* = 5)^a^	3 (*n* = 1)^a^	CTCAE reported	Yes	No	No	Yes	PRGS, RECIST
			Time frame (days)	NR				
			Grade 1-2	NR				
			Grade 3-4	NR				
			Grade 5	0				
Hilal et al. [31]	NR	NR	CTCAE reported	No	No	No	No	NR
Khomyakov et al. [24]	1 (*n* = 31)^a^	1 (*n* = 31)^a^	CTCAE reported	Yes^d^	-	Yes	Yes	PRGS
			Time frame (days)	30		30		
			Grade 1-2	3		3		
			Grade 3-4	1		1		
			Grade 5	0		0		
Khosrawipour et al. [32]	1.5 (*n* = 20)^a,b^	1 (*n* = 6)^a^	CTCAE reported	Yes^d^	Yes	Yes	Yes	TRG, PCI
			Time frame (days)	NR	NR	NR		
			Grade 1-2	6	3	3		
			Grade 3-4	0	0	0		
			Grade 5	1	1	0		
Nadiradze et al. [33]	2 (*n* = 24)^a^	NR	CTCAE reported	Yes^d^	No	No	Yes	Unspecified histological regression
			Time frame (days)	NR				
			Grade 1-2	15				
			Grade 3-4	7				
			Grade 5	2				
Reymond et al. [34]^a^	4 (3)^a^	7 (*n* = 1)^a^	CTCAE reported	Yes^d^	Yes	Yes	Yes	PRGS, RECIST
			Time frame (days)	NR	NR	NR		
			Grade 1-2	3	2	1		
			Grade 3-4	0	0	0		
			Grade 5	0	0	0		
Robella et al. [26]	3 (*n* = 14)^a^	3 (*n* = 13)^a^	CTCAE reported	Yes^d^	No	No	No	NR
			Time frame (days)	NR				
			Grade 1-2	14				
			Grade 3-4	0				
			Grade 5	0				
Farinha et al. [35]	NR	NR	CTCAE reported	No	No	No	No	NR
Hübner et al. [36]	NR	NR	CTCAE reported	No	No	No	No	NR

*CTCAE* Common Terminology Criteria for Adverse Events, *NR* not reported, *PCI* Peritoneal Cancer Index, *RECIST* Response Evaluation Criteria in Solid Tumors, *PRGS* Peritoneal Regression Grading Score, *TRG* Tumor Regression Grading

^a^median calculated from presented data

^b^mean reported in this publication

^c^reported per procedure

^d^reported per patient

Clavien Dindo (CD) classification was reported in two studies [30, 35], with a timeframe of either 30-days [35] or not specified [30]. The results were limited to statements saying that “…adverse events and surgical complications were mild, transient and self-limiting” [30] and that the “overall complication rate was 8.8%” [30].

Common Terminology Criteria for Adverse Events (CTCAE) data from 54 patients with information on treatment regimen (16 mono- and 38 bidirectional) showed that the proportion of patients experiencing an adverse event was 38% in the monodirectional and 21% in the bidirectional group – and these were almost exclusively grade 1–2 events (Table 4). Three studies reported a substantial number of CTCAE grade ≥ 3, but no information on the distribution between mono- and bidirectional treatment was provided [27, 28, 33].

OTR was reported using PCI, PRGS, TRG, RECIST or an unspecified histological regression model (Table 4). Four studies [24, 30, 32, 34] provided separate data on the bidirectional group, but these data were not homogenous enough to allow reporting and interpretation, and most of the patients received less than three PIPAC procedures before evaluation.

No included studies qualified for the evaluation of risk of bias in individual studies. The two prospective trials [24, 30] were evaluated for “outcome reporting bias” and found not to refer in their method section to a protocol or a clinical trial registration. Despite screening the International Clinical Trials Registry Platform (ICTRP), we were not able to match these studies to a protocol.

During screening for “positive publication bias”, we searched for unpublished trials through the ICTRP and the ISRTCN registries, using the search terms “PIPAC”, “pressurized chemotherapy” and “pressurised chemotherapy”. The search was carried out on the 6th of February 2019. Nineteen studies were identified through ICTRP and two through ISRTCN. One study on bidirectional treatment was identified, investigating repetitive ePIPAC and simultaneous IV chemotherapy for colorectal PM. The trial (EudraCT: 2017–000927-29) is still running and, therefore, could not be included in this review. Additionally, when screening the journal “Pleura and Peritoneum”, we identified two published protocols on ongoing PIPAC trials specifically evaluating the bidirectional approach in PM from upper GI- [37] (EudraCT: 2018–001035-40) and gastric cancer [38] (No registration number available).

No duplicate data were suspected among the included trials.

## Discussion

This systematic review shows that the bidirectional treatment approach is practiced and feasible but that no studies have attempted to compare it to either monodirectional PIPAC or to monodirectional systemic treatment. All studies were descriptive and showed a high degree of variation in terms of which outcomes were reported and how the outcomes were described. The studies were very heterogeneous including multiple malignancy types, different time-points of the diseases, varying disease extent and varying degrees of pre-treatment. Taken together, we were not able to draw any conclusions or perform a meta-analysis.

Regarding our main outcomes (OS, PFS and QoL), no comparisons between mono- and bidirectional treatment were possible - not even as simple additive analyses or narrative comments. Although 7 studies reported the overall survival using a similar definition (from the time of the first PIPAC procedure, practically no specific data on mono- vs. bidirectional treatment was published. PFS was not reported in any studies, while QoL data were reported in two studies, of which, only one provided actual data.

We chose the three outcomes that, in our view, are most important in assessing the potential benefits and harms of a bidirectional approach. A possible survival benefit due to the bidirectional treatment and to the presumable earlier initiation of PIPAC must be evaluated alongside its positive or negative impact on QoL. Our recommendation is that these outcomes should be standardized and included in any future PIPAC reporting.

We found a large variation in the reported PS criteria for patients entering PIPAC programs, but we were not able to evaluate PS specifically in the context of patients receiving bidirectional treatment. The median number of PIPAC procedures was similar in both the bidirectional and in the overall group, whereas the CTCAE reported adverse event rate was, surprisingly, lower in the bidirectional group. Theoretically, combining a surgical procedure with intravenous chemotherapy could lead to an increase in adverse events, thus, leading to a reduced number of PIPAC procedures – none of which were found. The data must be interpreted with great caution and unintentional selection of patients with good performance status for bidirectional treatment is a possible source of bias.

The reporting on complications and adverse events varied considerably across studies. One study [30] reported on surgical complications using Clavien-Dindo and on adverse events using CTCAE [30], while the remaining used either Clavien-Dindo [35] or CTCAE. When using CTCAE, eight of nine studies described this as a tool to report adverse events, but four of the studies then used the term “complications” in their CTCAE reporting in the results section. Another study [26] described that they used CTCAE to assess postoperative complications. Thus, it seems that the terms “adverse events” and “complications” are used interchangeably. Evaluation on whether an event is related to surgery (for which the Clavien-Dindo is intended [39]) or to the chemotherapeutic agent (where CTCAE is normally used) may prove difficult in a bidirectional treatment strategy. In the closely related field of CRS and HIPEC, Lehmann et al. showed that the interpretation of the severity of events between the two classifications differed with significantly higher major morbidity rates found using CTCAE compared to the Clavien-Dindo classification [40] – indicating that comparison between the two systems is not appropriate. Previous systematic reviews concluded that PIPAC directed therapy is associated with a low risk of complications and adverse events [1], but, in this review, data from the largest study with bi-directionally treated patients observed a significantly higher mortality and major complications rate than previously reported [27]. Despite its retrospective design it is important to note that these experienced PM centers had a learning curve regarding patient selection during the implementation of a PIPAC program, and that mortality and major complications occurred at the beginning of their experience. This observation was done in a mainly bi-directionally treated patient cohort and suggest that training in patient selection might lower the rate of adverse events.

OTR was reported using different definitions, and the majority of data were not specifically related to mono- or bidirectional treatment results. We recommend the components and use of OTR should be defined for future comparative trials.

We observed more women than men receiving bidirectional treatment in non-gynecological cancers, and the rate of bidirectional treatment varied between 10 and 100% in different cancer types. The latter variation may be influenced by multi-modal treatment regimens being more common in some cancer diseases, and the fact that larger PIPAC data are still limited on several indications. One third of the patients receiving bidirectional therapy did so without prior systemic chemotherapy. Considering the lack of evidence of efficacy and safety, on bidirectional PIPAC, this approach may seem premature. The use of PIPAC directed therapy as an early intervention in PM is the focus of several new studies, and a reduction in time from diagnosis of PM to the initiation of PIPAC may improve survival data.

The above findings should be interpreted with caution. The risk of possible biases is considerable since the studies were not designed to compare mono- with bidirectional treatment, the number of patients was small, and the method of reporting was not uniform. Secondly, a substantial part of the data on bidirectional patients came from one publication [24] treating all patients this way, and comparison with monodirectional patients was done across publications. Outcome reporting bias is also possible since no published protocol or registration in a clinical trial registry was found regarding the two prospective trials. We did not identify any unpublished trials concerning bidirectional PIPAC, and therefore, no positive publication bias was identified. The difficulty in drawing any conclusions based on this systematic review of the literature has highlighted the urgent need to improve and standardize reports on PIPAC directed therapy. We have, therefore, constructed a list of items to be considered when reporting on clinical PIPAC research (Additional file 1).

When examining the included studies, additional comments on bidirectional treatment were recorded in a non-formalized manner. The treatment-free window (no systemic chemotherapy) was scheduled to be between one and four weeks before PIPAC [26, 28, 31], and between zero and two weeks after the PIPAC procedure [26–28]. While the latter time frame is not relevant (using the present drugs for PIPAC), the use of some systemic drugs may prolong the treatment free interval in order to allow hematologic factors to normalize. The reported treatment intervals, however, and the need for a treatment-free window during bidirectional therapy are not based on scientific evidence. The bidirectional approach was performed on patient demand [28] and was, even though advised against by the PIPAC facility, independently sought by patients at outside institutions [31], indicating a patient-directed need for research on this treatment strategy. Bidirectional treatment was seen as an option to treat patients with PM and limited extraperitoneal disease, in which PIPAC as monotherapy was not indicated [26, 36]. Some studies interpreted their own data or referred to data from other institutions as promising evidence regarding the bidirectional approach [24, 28, 29, 32]. Some stated that PIPAC should enhance the efficacy of systemic therapy by reducing the intra-tumoural interstitial fluid pressure but without referencing this theory [26, 28]. Based on data in this review and the PIPAC literature, in general, the scientific evidence behind some of the statements is limited, and there is a risk of continuous citing of undocumented statements.

## Conclusion

Bidirectional treatment is practiced in many PIPAC centers and some patients are enrolled having received no prior systemic chemotherapy for their PM. Based on this systematic review we were unable to make any major conclusions stating whether bidirectional therapy is better or worse than PIPAC monotherapy. There is an urgent need for prospective trials focusing on bidirectional therapy and for consensus in determining how, when and which specific outcome variables should be reported.

We hope that this study, and the proposed list of items, will serves as a modest, but potential useful tool to help researchers improve international consensus on PIPAC reporting.

## Supplementary information

**Additional file 1.** Reporting items for PIPAC. List of items to be considered when reporting on PIPAC directed therapy.

## Data Availability

All data generated or analyzed during this study are included in this published article.

## Abbreviations

C/D: Cisplatin/Doxorubicin
CD: Clavien Dindo
CENTRAL: Cochrane Central Register of Controlled Trials
CTCAE: Common Terminology Criteria for Adverse Events
CUP: Cancer of Unknown Primary
ECOG: Eastern Cooperative Oncology Group
ePIPAC: Electrostatic Precipitation PIPAC
gem: Gemcitabine
HIPEC: Hyperthermic Intraperitoneal Chemotherapy
ICTRP: International Clinical Trials Registry Platform
IP: Intraperitoneal chemotherapy
IQR: Inter-Quartile range
IV: Intravenous
KI: Karnofsky Index
mito-c: Mitomycin c
nab-pax: Nab-Paclitaxel
NR: Not Reported
OS: Overall Survival
OTR: Objective Tumor Response
OXA: Oxaliplatin
PCI: Peritoneal Cancer Index
PFS: Progression Free Survival
PIPAC: Pressurized Intra-Peritoneal Aerosolized Chemotherapy
PM: Peritoneal Metastasis
PRGS: Peritoneal Regression Grading Score
PRISMA: Preferred Reporting Items for Systematic Reviews and Meta-Analyses
PROSPERO: International Prospective Register of Systematic Reviews
PS: Performance Status
QoL: Quality of Life
RECIST: Response Evaluation Criteria in Solid Tumors
TRG: Tumor Regression Grading
