# Supplementary material for: Bidirectional treatment of peritoneal metastasis with Pressurized IntraPeritoneal Aerosol Chemotherapy (PIPAC) and systemic chemotherapy: a systematic review
Source: BMC Cancer. 2020 Feb 10;20:105. doi: 10.1186/s12885-020-6572-6 (PMC7011374; doi:10.1186/s12885-020-6572-6)
Supplement: Supplementary file 1 — Additional file 1. Reporting items for PIPAC. List of items to be considered when reporting on PIPAC directed therapy. [file 12885_2020_6572_MOESM1_ESM.docx]

# Additional file 1: Reporting items for PIPAC

## List of items to be considered when reporting on PIPAC directed therapy

Based on the findings in our review we suggest a list of items that may be considered when reporting on PIPAC directed treatment. It is designed to be used for all types of PIPAC directed treatment regimens, thus including PIPAC as monotherapy and when PIPAC is performed in a bidirectional treatment plan in combination with systemic chemotherapy (SC).

The list includes variables that may be difficult to report in detail (e.g. type/dose/cycles of SC between PIPAC), and the list is only intended to provide basis for reflection when planning studies on PIPAC directed treatment.

To make reporting stringent and comparable we suggest that the starting point of the treatment course is set at the time of the first PIPAC, and that bidirectional treatment is initiated with a PIPAC procedure.

| **1. Patient** | |
| --- | --- |
| 1.1 Age | At the time of PM diagnosis |
| 1.2 Gender |  |
| 1.3 Primary tumor site (topography) | Term and topography code (e.g. ICD-10) |
| 1.4 Primary tumor in situ | Y/N |
| 1.4.1 Primary tumor resection | Date/month/year |
| 1.5 Primary tumor, histology | Term and morphology code (e.g. ICD-O-3) |
| 1.6 Diagnosis of PM | Date/month/year |
| 1.7 Time from primary tumor diagnosis to PM | Intervals (e.g. 0-2 months, 3-5 months,..) to avoid confusion of synchronous/metachronous definitions |
| 1.8 Metastasis outside the peritoneal cavity | Y/N |
| 1.8.1 Location of extra-peritoneal metastasis | Brain, lung, mediastinum, liver, retroperitoneum, bones, other. |
| 1.9 Previous oncological treatment (before the first PIPAC directed treatment) | Chemotherapy (type/dose/number of completed cycles)  Immunotherapy (type/dose/number of completed cycles)  Radiotherapy (dose/number of completed procedures)  Other |
| 1.10 Time from PM diagnosis to first PIPAC | (days) |
| 1.11 Performance status | ECOG, recorded immediately before the first PIPAC directed therapy |
| 1.12 Nutritional status | Anthropometric methods, recorded immediately before the first PIPAC directed therapy |
| **2. Staging** | |
| 2.1 staging modalities | CT/PET-CT/MRI (incl. assessment of retroperitoneal LNs)  Laparoscopy/laparotomy  Other |
| 2.2 PCI score | PCI may only be used for index stratification, since re-evaluation using present imaging modalities or laparoscopy is not possible |
| 2.3 Largest tumor nodule size | (long axis, mm, CT/MRI)  Probably same limitations as in 2.2 |
| **3. PIPAC directed and systemic therapy** | |
| 3.1 Treatment intention | Mono-/bidirectional  Palliative, Adjuvant, Downstaging |
| 3.2 Time from SC to PIPAC | Mean/median number of days from the last dose of systemic chemotherapy in a cycle to the following PIPAC procedure |
| 3.3 PIPAC chemo | Type/dose |
| 3.4 ePIPAC | Y/N |
| 3.4.1 ePIPAC - electrostatic precipitation time | (min) |
| 3.5 Non access |  |
| 3.6 Flow rate of infusion | (mL/s) |
| 3.7 Duration of procedure | (min) |
| 3.8 Diffusion time | (min) |
| 3.9 Ascites | (ml) |
| 3.10 PCI score | (see above) |
| 3.11 Systemic chemo between PIPAC | Type/dose/cycles |
| 3.12 Time from PIPAC to SC | Mean/median number of days from one PIPAC procedure to the first following dose of systemic chemotherapy |
| 3.13 Number of PIPAC procedures |  |
| 3.14 Interval between PIPAC procedures | (days) |
| **4. Complications/Adverse Event** | |
| 4.1 Complications/Adverse Events | (predefined list) |
| 4.1.1 Other Adverse Event |  |
| 4.2 CTCAE | 1-5 (CTCAE v5.0) |
| 4.3 Dindo-Clavien | I-V (30-day) |
| **5. Outcome** | |
| Objective Tumor Response (OTR) | Definition: Evaluation should include imaging and biopsies plus cytology after 3 PIPAC directed therapies |
| 5.1 Cytology |  |
| 5.2 PRGS |  |
| 5.3 Survival from first PIPAC directed treatment |  |
| 5.4 Survival from the diagnosis of PM |  |
| 5.5 QoL | Recorded at baseline immediately prior to first PIPAC and then after the third PIPAC |
| 5.6 Follow-up time |  |
| 5.7 Treatment program terminated | Y/N |
| 5.7.1 Reason for terminating treatment program | Complete remission (no evidence of PM on imaging, cytology and PRGS), progression, secondary non-access, patient wish, other |
